# Forcible Extension of Nerves in Traumatic Tetanus

**Published:** 1877-03

**Authors:** 


Forcible Extension of Nerves in Traumatic Tetanus.—Dr. Paul Vogt, of Griefswald, records (*Centralblatt fur die Chirurgie*, No. 40, 1876) an interesting case in which this proceeding was successfully performed. The patient was sixty-three years of age, and had injured his right hand by the falling of a stone. The ragged edges of the wound in the palm quickly united under the use of disinfectant dressing, but the dorsal surface healed very slowly. Fourteen days after the receipt of the injury symptoms of tetanus made their appearance. Large doses of opium and morphia, with local bathing, were adminministered, but violent attacks of opisthotonos and rigidity of the back and lower extremities supervened, followed by intercurrent clonic cramps. The wound three weeks after the accident presented a swollen cicatrix in the palm, and a granulating wound on the dorsum of the third metacarpal bone. Neither the cicatrix nor the wound was abnormally sensitive, nor was any pain produced by pressure on the nerves of the forearm or arm. The region of the brachial plexus was however very tender, and when pressure was made here, tonic contractions of the muscles of the neck followed. As the treatment adopted had proved ineffective, Vogt recommended division of the cicatrix and detachment of the edges of the wound, with neurectomy of the median and radial nerves, because the wound lay in the region of both nerves, and extension of the brachial plexus. This was carried out under the influence of an anæsthetic and with the employment of the carbolic-acid spray. After division of the cicatrix at the seat of the injury, a longitudinal cut was made at the anterior border of the trapezius, about two inches above the clavicle, the brachial plexus was exposed, raised with the index-finger, and the loops were drawn out and vigorously pulled in a centripetal and in a centrifugal direction. A drainage tube was inserted, and the wound was dressed with salicylicated jute. It should be mentioned that the nerve-sheaths, being red, were freely divided. On awakening from the narcosis the patient was found to be practically cured. The mouth could be readily opened, the tongue thrust out, and food swallowed. There were no remarkable alterations either of mobility or of sensibility in the arm. A violent but short attack of vomiting occurred twenty-four hours after the operation. Recovery was complete in a fortnight or a little longer.

